# Antireflection microstructures on ZnSe for mid- and far-IR fabricated by femtosecond laser ablation assisted with wet chemical etching

**DOI:** 10.1038/s41598-024-61191-3

**Published:** 2024-05-10

**Authors:** Andrei Teslenko, Tatiana Konstantinova, Andrey Bushunov, Artem Ibragimov, Ilya Rodionov, Mikhail Tarabrin

**Affiliations:** 1 Infrared Laser Systems Laboratory, Bauman Moscow State Technical University, Moscow, Russia 105005; 2 FMN Laboratory, Bauman Moscow State Technical University, Moscow, Russia 105005; 3 Dukhov Automatics Research Institute, VNIIA, Moscow, Russia 127030

**Keywords:** Solid-state lasers, Design, synthesis and processing, Surface patterning, Laser material processing, Nonlinear optics, Optoelectronic devices and components

## Abstract

Most infrared materials used in high-power systems, such as optical parametric generators, have high values of refractive indices, which result in high Fresnel losses. The performance of conventional antireflection coatings is limited when used in high-power and ultra-broadband systems. An alternative approach is to fabricate antireflection microstructures (ARMs) that allow for a broadband increase in transmittance without reducing the damage threshold of the material. In this work, ARMs were fabricated on the surface of ZnSe crystals using the femtosecond laser ablation assisted with wet chemical etching method. This allowed to produce high aspect ratio microstructures that increase the transmittance up to 98% in the mid- and far- infrared regions.

## Introduction

ZnSe is an infrared optical material that is transparent in a wide spectral range (0.45 to 21 µm)^1^, has an average refractive index of 2.43 in the 0.45–21 µm range, and a large bandgap (around 2.7 eV)^2^. All of this results in the use of ZnSe in various optoelectronic^3^ and medical devices^4,5^ as well as infrared optics. However, due to the relatively high refractive index, Fresnel reflection losses reach around 18% per surface, which negatively affects the energy performance of the aforementioned systems.

The conventional method of decreasing reflection implies the use of dielectric antireflection coatings (ARCs). However, this technology has several disadvantages. For instance, ARCs have relatively low laser-induced damage threshold (LIDT) values compared to the LIDT values of the untreated material^6^. This limits their usage in high-power and high-energy optical systems, such as lasers and widely tunable optical parametric generators for mid- and far-IR^7–9^. Furthermore, creating an operational broadband ARC for a wide range of incidence angles is a complicated task^10^.

An alternative method is to fabricate antireflection microstructures (ARMs) on the surface of optical elements^11^. The ARM can be described as a system of periodic cavities or protuberances with a period ranging from several hundred nanometers to several micrometers. The physics that underlies the operational principle of the ARM can be simply described by effective medium theory^12^ which represents a composite material as an effective medium with a set of constitutive parameters. The shortest wavelength in the ARM operating range is the wavelength that allows incident radiation to interact with the ARM as if it were a gradient index layer. In this case, the radiation passes without encountering an abrupt border between media, which, in turn, reduces Fresnel reflection^13^. The interaction of light with the ARM in this mode does not cause significant light scattering^14^. This wavelength can be found as $\lambda _{threshold} = n \cdot p$ where *n* is the refractive index of the material and *p* is the microstructure period. The expression is derived from a simple diffraction grating equation where the first-order diffraction angle is considered to be 90 °.

The ARM enables the enhancement of transmittance across a wide range of wavelengths^15,16^. In addition, it has a higher LIDT^6,17^ compared to ARCs and does not depend on the adhesion of the optical surface. This enables the utilization of ARMs in high-energy optical systems, even with materials that are not suitable for proper polishing or have issues with adhesion^18–20^. Furthermore, the ARM is capable of operating at a wide range of incidence angles without significant transmittance losses^21^.

In recent years, different methods for creating ARMs have been developed^11,16^. One of the most common methods for creating such microstructures is reactive ion etching (RIE), the so-called dry etching method, where the mask for RIE is produced beforehand using interference lithography ^22^. Although ARMs produced by this method have been proven to achieve high transmittance values, the method itself has some crucial drawbacks. For example, several complex technological steps are required, which makes the method both costly and time-consuming. In addition, the reactive ion etching process requires a clean chamber for each material, which significantly increases the cost. Furthermore, a common problem with masking techniques is that some substrates have poor surface quality for mask deposition, such as photoresists or colloid solutions. Therefore, they can only be treated using maskless dry etching methods. It is known that when maskless dry etching techniques are used, random ARMs (RARMs) can be produced^23^. Although these types of ARMs can increase transmittance up to 99.9% in the 1–2 µm wavelength range^23^, it is much harder to control their morphology. This results in technological issues for fabrication in mid- and far- IR: for longer wavelengths, RARM starts to act as a rough surface, limiting the maximum transmittance.

One of the more straightforward methods for the fabrication of periodic ARM is the femtosecond laser ablation method. It is based on local material removal by femtosecond laser pulses. This method requires only a single technological step and is more cost-effective while maintaining relatively high throughput^24^. However, the use of a Gaussian beam does not allow the fabrication of high-aspect-ratio ARMs and therefore limits the maximum achievable transmittance^16^. Some groups have tried using Bessel beams to increase the depth of the ARM^25–27^, but creating a high-quality Bessel beam is not a trivial task, which resulted in the limited ARM performance fabricated with this method.

Another drawback of the method is the ablation residue, which condenses on the surface of the sample, decreasing the transmittance due to scattering and diffraction. To counteract this effect, in our previous work we tried implementing a constant supply of dried and filtered compressed air directed to the ablation zone^18^, however, the compressed air affected only some of the evaporated material, reducing the amount of contamination, but not completely removing the residue.

Therefore, to achieve the highest transmittance, two issues must be addressed: increasing the depth of the ARM and the fill factor without destroying the walls between cavities and removing the ablation residue. Wet chemical etching is one of the ways to form deep microstructures and ensure smooth surfaces^28^.

In this work, we demonstrate an approach that involves wet etching as an additional technological step after femtosecond laser ablation. The effect of several chemical treatments on the transmittance and surface morphology of ZnSe was analyzed using Fourier spectroscopy and scanning electron microscopy. The proposed method allowed us to increase the maximum transmittance up to 98% at 9.2 µm and the average transmittance up to 93.2% in the 6–18 µm wavelength range.

## Experiment

**Figure 1:**
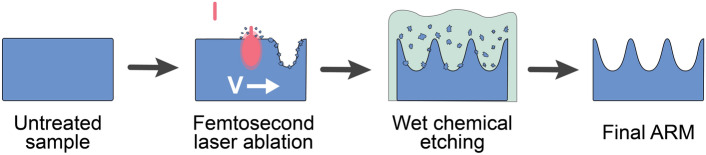
Schematic diagram of the experimental steps.

The schematic diagram of the experiment is demonstrated in Fig. 1. A detailed description of each step is provided later in this section.

### Fabrication of ARM

For ARM fabrication, a femtosecond laser ablation method was used. It is based on local material removal by high-intensity femtosecond laser pulses. By controlling the energy distribution in the cross-section of the laser beam, the movement velocity of the sample, and the laser repetition rate, one can produce ARMs of different morphology. To additionally vary the depth, the number of pulses per cavity can be altered. However, it is necessary to take into account the ”smearing” of the laser pulses along the sample movement direction. This factor limits the maximum amount of pulses per cavity, as the mentioned effect can negatively affect the ARM morphology by destroying the walls between the cavities. This effect can be countered if, instead of constantly moving the sample, it is stopped every time a pulse burst is sent. However, this increases the production time manifold, therefore this method is not considered in this paper.

A Pharos Yb:KGW laser (Light Conversion, Lithuania) was used as the femtosecond pulse source for ARM fabrication. The maximum amount of available power at the operational wavelength (513 nm) is 1.5 W or 0.2 mJ of pulse energy. For sample positioning, Aerotech ANT-90 (Aerotech Incorporated, USA) three-axis nanopositioners were used to provide in-position stability of 2 nm and repeatability of 75 nm at velocities of up to 200 mm/s. A 100 × objective lens (Mitutoyo Corporation, Japan) was used to focus the laser beam on the sample surface.

To fabricate all the ARM samples, the base repetition rate was set to 200 kHz, however, the system is equipped with an additional pulse picker to reduce the repetition rate in correspondence with the sample movement velocity and the required period of ARM. The number of pulses per cavity was chosen experimentally and for all the samples amounted to 20. For a period of 2.3 µm and a sample velocity of 5 mm/s the additional pulse picker was automatically set to approximately 2.2 kHz. This means that a packet of 20 pulses with a repetition rate of 200 kHz between individual pulses was sent with a repetition rate of 2.2 kHz to form the ARM period of 2.3 µm. The average laser power varied from around 20 to 60 mW, the pulse energy values were therefore changed from 0.1 µJ to 0.3 µJ.

The laser pulses were focused somewhat below the surface of the sample. The so-called z-shift was introduced based on our previous findings^24^ and allowed us to produce ARMs with increased depth. While the starting values of most parameters were chosen based on visual feedback from a microscope implemented in the system, fine-tuning of the z-shift, average power, and ARM period was performed based on the transmittance measurements. To remove residual ablative material and other debris from the surface of the sample, a constant supply of dried and filtered compressed air directed to the ablation zone was used. The air pressure was experimentally adjusted so that it did not disturb the ablation process.

ARM measurement of spectral characteristics was performed with a Bruker Lumos Fourier transform IR spectrometer (Bruker Corporation, USA). Each measurement was averaged over 32 scans, the aperture size during a single point measurement was 100 × 100 µm, whilst the spectral resolution was set to 4 cm^-1^. The measurement was performed in the following order. First, using the same spectrometer, the reflectance of an untreated surface was measured and averaged over 100 points on the sample. Then, the transmittance of the ARM was measured relative to the transmittance of the untreated surface (the untreated surface transmittance was set as a background measurement). It is important to note that the background was measured every time before the actual sample to minimize the time between the two measurements, thus reducing the error resulting from fluctuations of pressure and temperature in the surrounding environment. Then, a single-surface transmittance for the ARM was calculated. However, the setup does not allow for chamber evacuation, thus some features of air and water vapor absorption can be observed when analyzing the data.

After fabrication, the morphology of each ARM was analyzed using a scanning electron microscope (SEM). For cross-sectional analysis, the samples were split into half using a femtosecond laser and a glass cutter. Besides, the transmittance of each sample was measured and recorded. Later on, this data will be referred to as the ”before-etching transmittance”.

**Figure 2:**
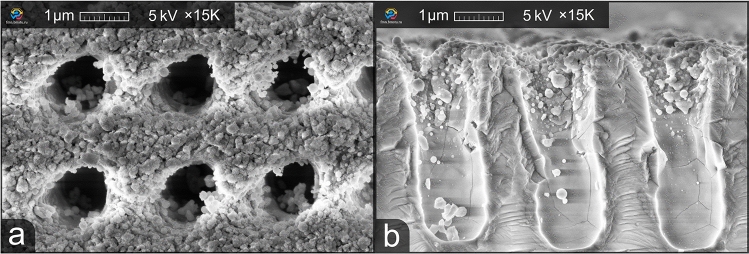
Top-down (**a**) and cross-section, (**b**) views of the fabricated ARM before etching.

### Wet chemical etching

Femtosecond laser pulse irradiation focused on the ZnSe wafer induces stress, structural changes, and amorphization on the substrate surfaces. The combination of femtosecond laser ablation and etching technologies enables mask-free fabrication of antireflection microstructures.

There have been reported several wet etching methods for ZnSe crystal chemical treatment. For example, HCl^25^, HNO_3_, H_2_SO_4_^29^, K_2_Cr_2_O_7_, and KMnO_4_-based solutions^30^ have been reported to be suitable chemical etchants for removing surface damage; however, they left a relatively smooth surface covered by an amorphous Se layer. Basic solutions (NH_4_OH, NaOH) treatment demonstrated that this solution successfully removed the oxide overlayer of ZnSe^31,32^. Some preliminary experiments were carried out and the difference in surface conditions was observed when HNO_3_ acid-base and NH_4_OH–alkaline solutions were used.

After laser irradiation, it was critical to find an effective way to selectively and efficiently deepen the laser-treated area and increase the fill factor of the ARM . Several solutions described above were tested during preliminary experiments; however, they did not prove to be efficient. In this work, the use of two main etching solutions is described. The first solution is a mixture of nitric, acetic, and orthophosphoric acids (Al etching, Type A, Transene Company Inc.) (solution A). The second one is 1:1:1 NH_4_OH (29%):H_2_O_2_(30%):HO_2_O solution (solution B). The best results were achieved during the process carried out at room temperature for a period of 3 mins and 5 mins, respectively. The use of an aqueous alkaline solution leads to a reduction of the etching rate and prevents the destruction of ARM’s walls. All chemicals were of VLSI grade. All samples were immediately rinsed with deionized water and dried in N_2_ gas after etching.

**Figure 3:**
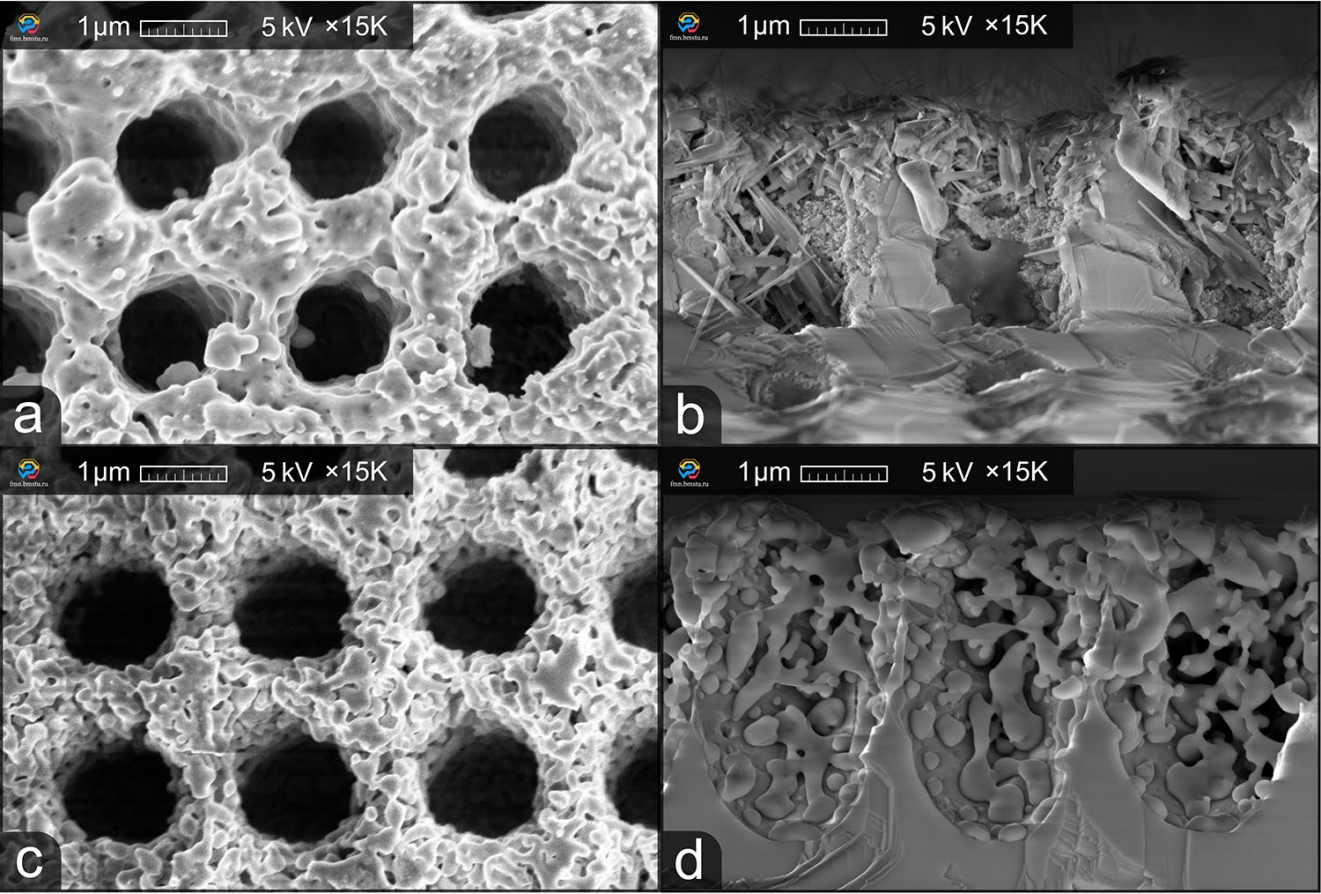
Top-down and cross-section views of solution A (**a**, **b**) and solution B (**c**, **d**) etching effect on the ARM.

## Results and discussion

### Before-etch ARM

A total of 400 ARMs with different parameters were fabricated. The transmittance of all samples was measured and recorded. Identical 3 × 3 mm^2^ samples with the best transmittance were selected for further etching. Each sample was cut in half to perform a preliminary morphological analysis using SEM before etching. The top-down view and the cross-section view of one of the samples are shown in Fig. 2.

It can be seen that the ARM is periodic, the period is 2.3 µm while the height is around 3.8 µm. This provides an aspect ratio of 1.7. However, as can be seen in Fig. 2, the fill factor is around 0.75, which limits the maximum transmittance. In addition, a significant amount of ablative residue is present on the surface, which can negatively affect the transmittance by scattering incident light.

### After-etch ARM

Figure 3 shows an image of the two samples obtained after etching in two different solutions: solution A (etched for 3 minutes) for Fig. 3a,b and solution B (etched for 5 minutes) for Fig. 3c,d. It can be seen that the solutions demonstrate a different effect on the microstructures. To confirm the structural and chemical changes involved in the etching processes, it is necessary to understand the chemical reactions.

Solution A acts on ZnSe by H_3_PO_4_ and HNO_3_ polishing of Zn and oxide overlaying formation on the ZnSe crystal^33^. Etching proceeds according to Eq. (1)^29^:

1 $$\begin{aligned} 3\hbox { ZnSe} + 8\hbox { HNO}_{3} -> 3\hbox { Se} + 2\hbox { NO} + 4 \hbox {H}_{2}\hbox {O} + 3\hbox { Zn}{(\hbox {NO}_{3})}_{2} \end{aligned}$$

**Figure 4:**
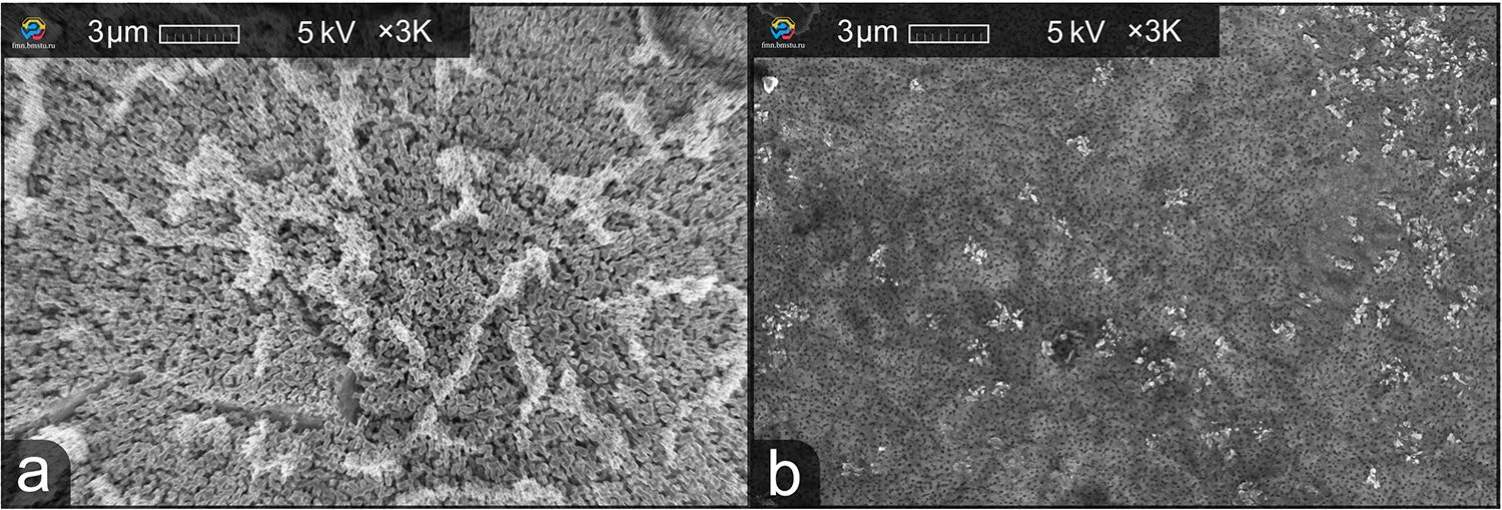
Top-down view of the 500-nm-deep ARM after a prolonged etching in solution A (**a**) and additional etching in HF-based solution (**b**).

The needle-shaped whiskers were observed by SEM in the cross-section view of the etched microstructures. The increased amount of Se and Zn oxide overlayer amount on the surface can lead to the formation of nanowhiskers, which can also be observed in the literature^34,35^. It is shown in the literature that the oxide overlayer can be removed with a HF-based solution. This was noticed after etching one of the solution-A-etched ARM samples in HF-based solution for 10 minutes. It was an ARM sample with a depth of 500 nm, and as a result of prolonged (8 minutes) etching in solution A, the structures were completely wiped out and a Se-rich layer was formed on the surface of the sample (Fig. 4). Etching in HF solution was performed after a month of aging. We assume that during this time selenium oxide was formed and then removed in hydrofluoric acid solutions. This observation can be used in future work to remove the deposited layer without damaging the ZnSe crystal.

Solution B modified the surface to a sponge-like porous structure after etching (Fig. 3b). NH_4_OH solution does not etch the ZnSe itself^31^. In this solution H_2_O_2_ acts as an oxidizing agent (2)^36^:

2 $$\begin{aligned} \hbox {ZnSe} + 2\hbox { H}_{2}\hbox {O}_{2} {\mathop {\longrightarrow }\limits ^{{\text{NH}}_{4}{\text{OH}}}} \hbox {ZnO}_2 + \hbox {Se} + 2\hbox { H}_{2}\hbox {O} \end{aligned}$$

In total, it can be observed in Fig. 3d that the fill factor of the microstructure after etching in solution B increased to a value of approximately 0.9 — the walls became thinner and the diameter of the cavities increased. Furthermore, the transition between the vertical and horizontal walls became smoother, thus forming a smoother refractive index gradient . However, the height decreased to around 3.4 µm. This occurred due to the fact that wet chemical etching is an isotropic process, which means that it is impossible to selectively deepen the ARM using this method. However, an increase in the diameter of the crater resulted in an increase in the fill factor, which positively affected the ARM transmittance. This will be addressed in more detail below.

### Ultrasonic cleaning

It is important to note that both solutions resulted in the formation of a red film on the ZnSe surface. The etch predominantly removed Zn, leaving a red Se-rich surface with a predominantly Se-Se bonding^37^. We assume that the droplets formed on the surface of the samples and in the structures could be a selenium film mentioned in the literature. This thin film has low adhesion to the surface; therefore, to remove sedimented Se on the surface of the sample, an ultrasonic (US) cleaning technique can be used^34^.

**Figure 5:**
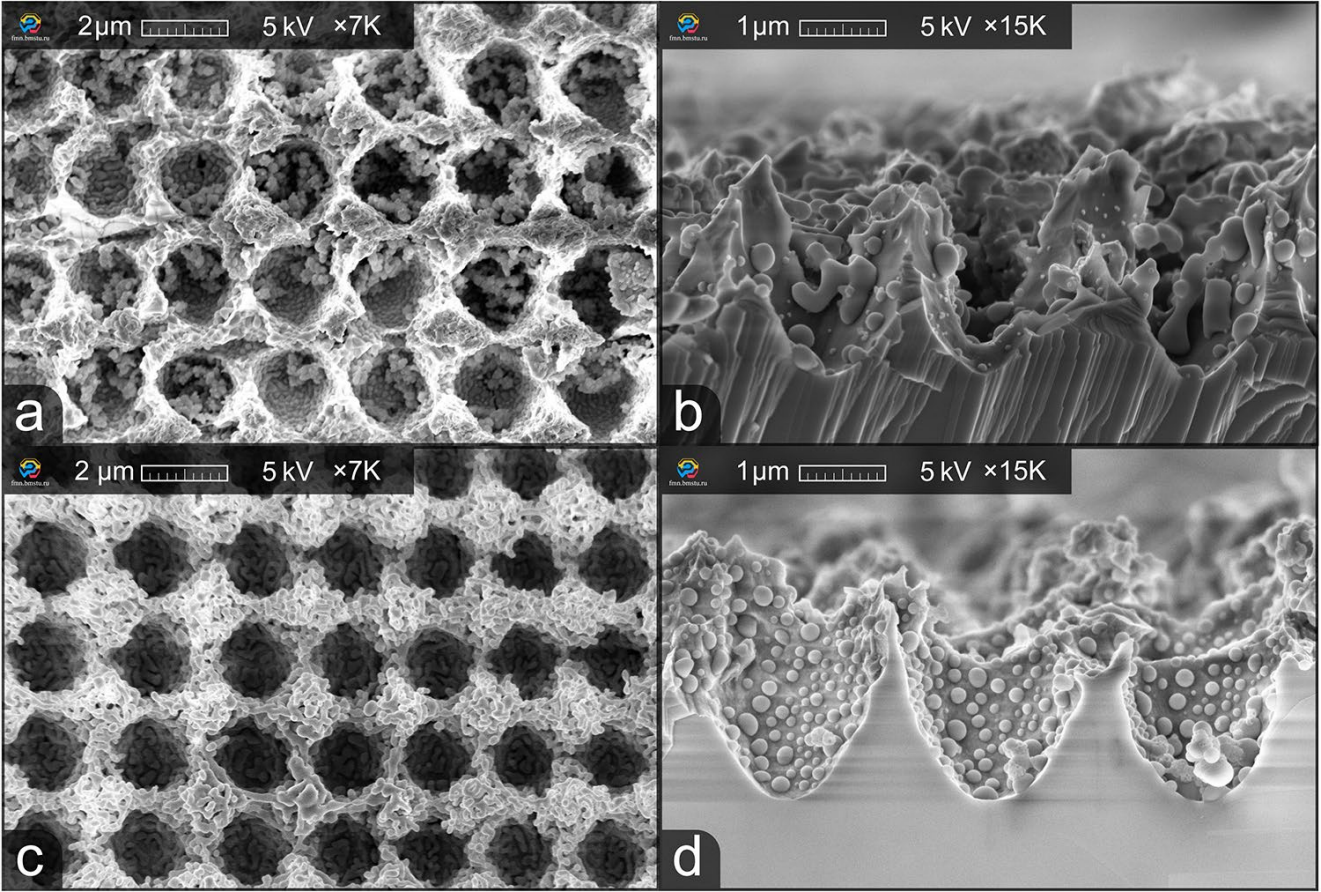
Ultrasonic damage of ARM during etching in solution B and consequent rinsing: top-down (**a**), cross-section (**b**); and during rinsing: top-down (**c**), cross-section (**d**).

There are two ways of using ultrasonic cleaning: during an etching stage to prevent the deposition of Se film or during a water rinsing stage after etching to remove the film. Elma P30H ultrasonic bath (Elma Schmidbauer GmbH, Germany) was used for the process. Frequency of 80 kHz with different ultrasonic power (30–100% of 120 W) was used for film removal. Figure 5 shows the effect of using US cleaning. Ultrasonic agitation during etching and subsequent rinsing (ultrasound was applied during both stages) leads to a reduction in the deposition of the reaction product (Fig. 5a,b). When ultrasonic treatment is used only during rinsing, it removes the Se layer just partially (Fig. 5c,d).

It can be seen that both cases significantly damage the ARM. High-intensity sound waves generate pressure fluctuations that result in the formation of cavitation bubbles and uneven sonic impact across the sample. Due to this, the use of ultrasonic agitation by 80 kHz during etching or rinsing is not a suitable method for removing reaction products. Higher frequencies of ultrasound could potentially yield better results as the cavitation bubbles should be smaller in size compared to the dimensions of the individual cavities. In addition, the literature suggests that the Se layer could be removed by etching in CS_2_^30^.

### Transmittance measurement and numerical simulation

**Figure 6:**
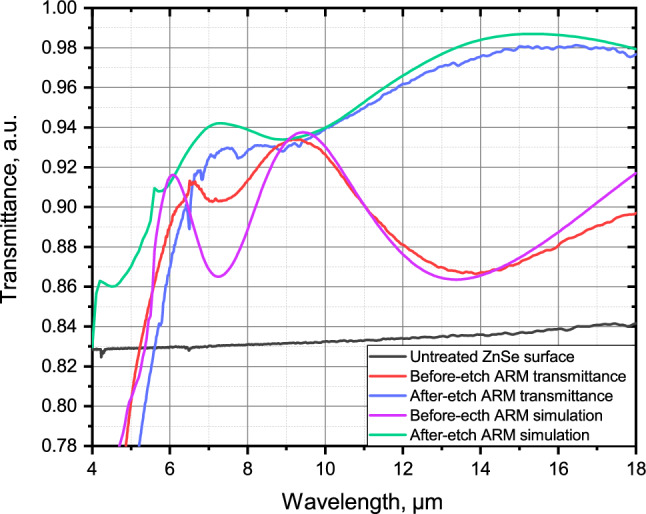
Transmittance of the best ARM before and after etching in solution B compared to the transmittance of the untreated surface along with before-etch and after-etch ARM simulations.

The spectra of all the fabricated ARMs were measured using a method described above. Figure 6 shows a one-side transmittance of the best ARM measured before and after etching compared to the transmittance of the untreated surface; material absorption is not taken into account. This sample, an image of which is shown in Fig. 3c and d, was etched in solution B for 5 minutes without ultrasonic agitation during either etching or rinsing stages. The transmittance was averaged over 25 points on the sample surface, each point averaged at 50 scans. The before-etch maximum transmittance of 93.4% at 9.2 µm was increased to the maximum transmittance of 98% at 16 µm after etching, therefore increasing the average transmittance in the 6–18 µm wavelength range from 89.8% before etching to 93.2% after etching. This is almost 10% higher than the average transmittance of an untreated ZnSe sample in the 6–18 µm wavelength range.

The sinusoidal character of ARM transmittance before etching occurs due to the formation of an equivalent $\lambda /4$ layer^38,39^. 20 pulses were used to form a single cavity, therefore flattening the bottom of the crater and creating cavities with a cylinder-like cross-section profile. The etching process helped to smear these cavities, simultaneously creating a pronounced gradient-index layer, making the equivalent layer thinner and moving the transmittance peak towards shorter wavelengths.

Figure 6 also demonstrates numerical simulations of the transmittance for microstructures with different morphology. For that, a commercially available finite element modeling package was used. The modeling setup was a three-dimensional domain with an emitter port and a receiver port, with a single microcavity placed in between. A more detailed description of the setup can be found in our previous work^18^.

The before-etch ARM transmittance was simulated with the following morphological parameters: period of 2.3 µm, height of 3.7 µm, and fill factor of 0.75. The transmittance in the 6.5–9 µm range for the real ARM is higher due to imperfections in the $\lambda /4$ layer which results in smaller-amplitude modulations.

The after-etch ARM transmittance was simulated with the following morphological parameters: period of 2.3 µm, height of 3 µm, and fill factor of 0.86. The transmittance peaks at 7 and 16 µm correspond to the measured transmittance peaks. This means that the height of the equivalent $\lambda /4$ layer decreased after etching, therefore shifting the peaks towards shorter wavelengths. Additionally, the fill factor increased significantly, resulting in higher overall transmittance values. However, the transmittance in the short-wavelength range (4–6 µm) for the measured ARM is smaller than for the modeled one. This can be explained by diffraction and scattering that occur at shorter wavelengths due to the residual material created by wet chemical etching (Fig. 3d).

## Conclusion

In this work, we demonstrated an approach for ARM fabrication in the mid-IR, involving multipulse femtosecond laser ablation followed by wet chemical etching. This allowed to increase the average transmittance in the 6–18 µm wavelength range from 83.5% for the untreated surface to 93.2% for the ARM-treated surface, providing a maximum transmittance of 98% at 16 µm. This can be used for high-power parametric systems in the mid- and far-IR.

In our future work, we plan to investigate the use of higher-frequency ultrasound for Se film removal, as well as etching in CS_2_ solution. This might help increase the transmittance of the fabricated ARM even more.

## Data Availability

Measurement data are available on request by email from the corresponding author.
